# Radiation exposure and mortality risk from CT and PET imaging of patients with malignant lymphoma

**DOI:** 10.1007/s00330-012-2447-9

**Published:** 2012-04-27

**Authors:** R. A. J. Nievelstein, H. M. E. Quarles van Ufford, T. C. Kwee, M. B. Bierings, I. Ludwig, F. J. A. Beek, J. M. H. de Klerk, W. P. Th. M. Mali, P. W. de Bruin, J. Geleijns

**Affiliations:** 1 Department of Radiology, Medical Center Haaglanden, The Hague, The Netherlands; 2 Department of Radiology (E 01.132), University Medical Center, P.O. Box 85500, 3508 CX Utrecht, The Netherlands; 3 Department of Pediatric Hematology, University Medical Center, Utrecht, The Netherlands; 4 Department of Hematology, University Medical Center, Utrecht, The Netherlands; 5 Department of Nuclear Medicine, Meander Medical Center, Amersfoort, The Netherlands; 6 Department of Radiology, University Medical Center, Leiden, The Netherlands

**Keywords:** Radiation exposure, Medical imaging, Malignant lymphoma, Computed tomography, Positron emission tomography

## Abstract

**Objective:**

To quantify radiation exposure and mortality risk from computed tomography (CT) and positron emission tomography (PET) imaging with ^18^F-fluorodeoxyglucose (^18^F-FDG) in patients with malignant lymphoma (Hodgkin’s disease [HD] or non-Hodgkin’s lymphoma [NHL]).

**Methods:**

First, organ doses were assessed for a typical diagnostic work-up in children with HD and adults with NHL. Subsequently, life tables were constructed for assessment of radiation risks, also taking into account the disease-related mortality.

**Results:**

In children with HD, cumulative effective dose from medical imaging ranged from 66 mSv (newborn) to 113 mSv (15 years old). In adults with NHL the cumulative effective dose from medical imaging was 97 mSv. Average fractions of radiation-induced deaths for children with HD [without correction for disease-related mortality *in brackets*] were 0.4% [0.6%] for boys and 0.7% [1.1%] for girls, and for adults with NHL 0.07% [0.28%] for men and 0.09% [0.37%] for women.

**Conclusion:**

Taking into account the disease-related reduction in life expectancy of patients with malignant lymphoma results in a higher overall mortality but substantial lower incidence of radiation induced deaths. The modest radiation risk that results from imaging with CT and ^18^F-FDG PET can be considered as justified, but imaging should be performed with care, especially in children.

**Key Points:**

- *Survival of malignant lymphoma has improved dramatically over the past decades*.
- *PET and CT currently play important roles for malignant lymphoma patients*.
- *The potential hazard of ionising radiation has become an increasingly important issue*.
- *When assessing radiation risks, disease-related reduction in life expectancy should be considered*.
- *CT and* ^*18*^ *F-FDG PET create a modest radiation-induced mortality risk*.

## Introduction

Imaging plays an important role in the management of patients with malignant lymphoma (Hodgkin’s disease [HD] and non-Hodgkin’s lymphoma [NHL]). Accurate imaging is important for determining the stage of disease, which guides the treatment strategy and influences the prognosis [1]. Imaging is also important for assessing response to therapy and detecting tumour persistence or recurrence [2–4]. Computed tomography (CT) and, more recently, positron emission tomography (PET) with ^18^F-fluorodeoxyglucose (^18^F-FDG) have become indispensable tools in oncological imaging [2, 5–9]. However, CT and ^18^F-FDG PET result in exposure of the patient to ionising radiation, which is associated with a carcinogenic risk [10–17].

There is a worldwide growing concern about radiation exposure in medical imaging [12, 18–21]. Estimates of the National Council on Radiation Protection and Measurements (NCRP) Scientific Committee in the United States in 2006 show an almost sixfold increase in the per capita dose from medical exposure to about 3 mSv compared with 1982. CT and nuclear medicine examinations are the largest contributors [22].

Therapeutic advances have dramatically improved the survival of patients with malignant lymphoma over the past decades. HD can nowadays be cured in at least 80% of patients [23–25]. Therefore, current treatment strategies not only aim at maximising curative success but also at minimising (late) toxicity, such as infertility, premature menopause, cardiac disease, and most importantly, risk of second neoplasms [24, 25]. In this context, the potential hazard of ionising radiation that is associated with diagnostic CT and PET in patients with malignant lymphoma is an increasingly important issue. Therefore, performing dose and risk assessment should be prioritised for circumstances with high cumulative radiation exposure for individual patients and better survival rates. This is particularly true when patients are young, such as children with HD, and when the cumulative radiation dose from imaging is expected to fall within the range of 5–150 mSv, such as during follow-up of patients treated for malignant lymphoma [10–13, 15]. This latter dose range is based on the studies in the subgroup of atomic bomb survivors who received low doses of radiation, ranging from 5 to 150 mSv (mean dose of 40 mSv), which suggested a significant increase in the overall risk of cancer in this subgroup [14–16].

The aim of this study was to perform accurate radiation risk assessment for imaging of patients with malignant lymphoma. To achieve our goal, the radiation exposure of CT and ^18^F-FDG PET was calculated for a typical work-up for children with HD in different age categories, and for adults with NHL. We expected that disease-related mortality would have a significant effect on the assessment of radiation risk in patients with malignant lymphoma, and that disease-related mortality would be much higher compared with radiation-induced mortality. This expectation is in accordance with a very recent publication by Brenner et al. [26]. Advanced radiation risk assessment, based on the demographic methodology of life tables, was developed to take into account these effects.

## Materials and methods

### Clinical practice of imaging

Malignant lymphoma comprises a heterogeneous group of diseases, differing with regard to histology, treatment and outcome. It is beyond the scope of this study to encompass all the different entities. Instead we focus on the most common types of malignant lymphoma; i.e. HD in children and diffuse large B-cell lymphoma (DLBCL) in adults [27–30]. The most typical imaging strategy for children with HD (age <18 years) and for adults with DLBCL was used in this study. In children with HD, the imaging strategy was based on the protocol according to the Children’s Oncology Group [COG]; in adults with DLBCL, the imaging strategy was based on the HOVON 84 international multicentre trial currently running in The Netherlands [www.hovon.nl, EudraCTnr. 2006-005174-42] (Table 1).

**Table 1:** The imaging strategy for children with Hodgkin’s disease (*HD*) and adults with diffuse large B-cell lymphoma (*DLBCL*)

	Children with HD	Adults with DLBCL
Most common group	Intermediate Risk Strategy: stage I-A bulky disease, I-AE, IB, II-A bulky disease, II-AE, II-B, III-A, III-AE, III-AS, III-AE+S, IV-A en IV-AE	Stage II–IV
Initial diagnostic	1 CT of neck-chest-abdomen	1 CT of neck-chest-abdomen
	1 whole-body ^18^F-FDG PET	1 chest X-ray
	1 chest X-ray	
Therapeutic phase	2 CTs of neck-chest-abdomen	2 CT scans of neck-chest-abdomen
	1 whole-body ^18^F-FDG PET (directly after therapy)	1 whole-body ^18^F-FDG PET scan
	2 chest X-rays	
Follow-up	2 CTs of neck-chest-abdomen	4 CT scans of neck-chest-abdomen
	7 CTs of neck and chest (for most common supradiaphragmatic stage I and II disease)	2 chest X-rays
	4 chest X-rays	
Timing of CT during follow-up	At 3, 6, 9, 12, 15, 18, 24, 36 and 60 months	At 6, 12, 18 and 24 months

Imaging with ultrasound and chest radiography were not considered in this study, as ultrasound is not associated with radiation exposure, and the radiation exposure from chest radiography is negligible compared with CT and ^18^F-FDG PET.

### Radiation exposure from CT

**Fig. 1:**
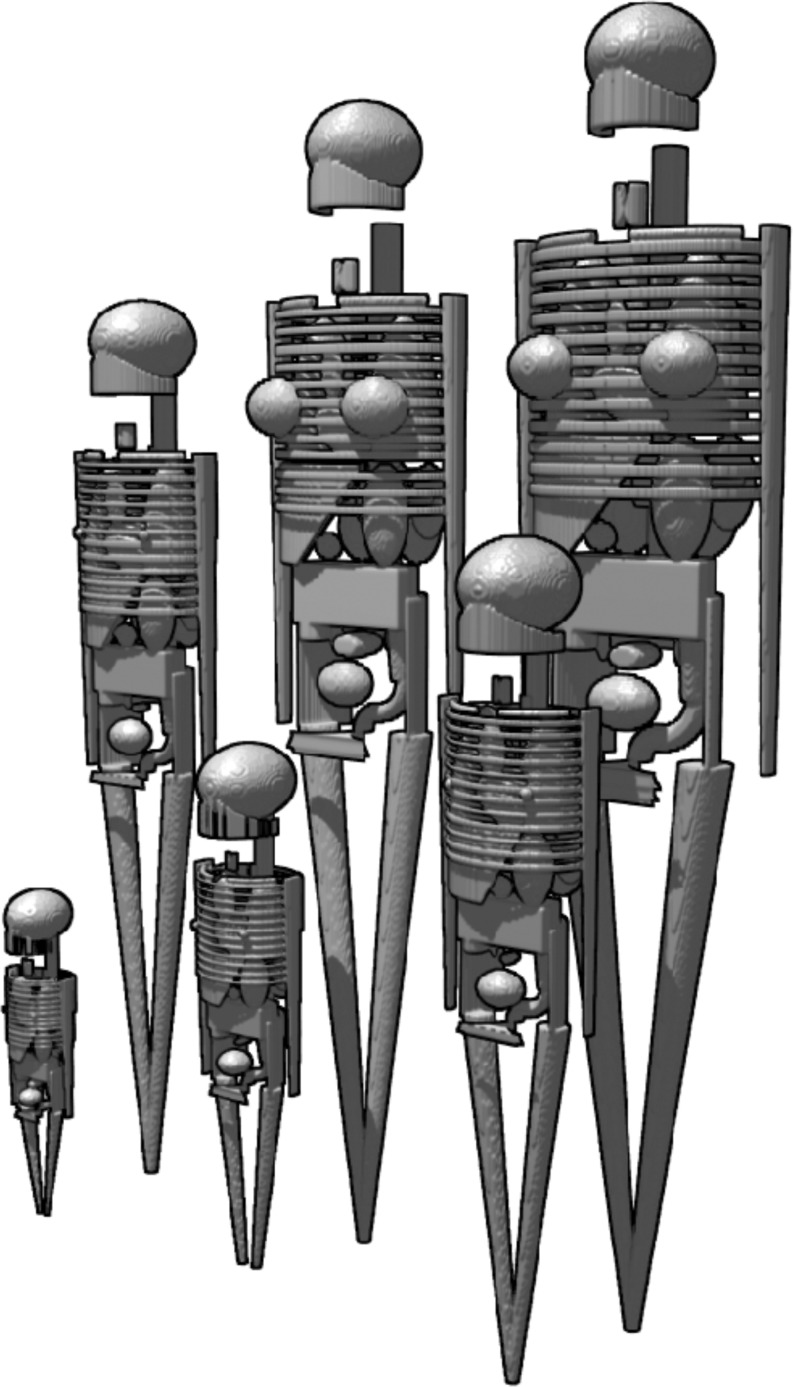
The mathematical hermaphrodite Medical Internal Radiation Dose (MIRD) patient models; their body, skeleton and organs are described by spheres, ellipsoids and cones. The paediatric patient models are divided into five categories according to their age and weight: newborn (3.6 kg, *left first row*); 1 year old (9.7 kg); 5 years old (19.8 kg); 10 years old (33.2 kg); 15 years old (56.8 kg). The adult model represents an average-sized patient of 74 kg (*right second row*)

There was no information available that allowed for assessment of organ doses in paediatric patients undergoing CT. Therefore we created Medical Internal Radiation Dose (MIRD) paediatric patient models in five general categories according to age and weight: newborn (3.6 kg), 1 year old (9.7 kg), 5 years old (19.8 kg), 10 years old (33.2 kg) and 15 years old (56.8 kg). The adult MIRD patient model represents an average-sized patient of 74 kg. All these hermaphrodite patient models (MIRD V) are described by spheres, ellipsoids and cones, as illustrated in Fig. 1 [31]. For assessment of radiation exposure from CT, we used these patient models in combination with an algorithm for Monte Carlo dose calculations (ImpactMC software, version 1.0, VAMP, Erlangen, Germany) [31, 32]. The Monte Carlo dose calculations are based on a virtual model of the CT system with respect to geometry, X-ray spectrum, filtration and CT parameters. The simulation is performed on 3D voxelised versions of the MIRD V phantoms. To each voxel in the volume during the simulation, a density value and a mass attenuation coefficient are assigned corresponding to five different materials: air, lung, soft tissue, fat and bone. During the simulation of a CT examination, the energy deposited in each voxel (absorbed dose) is accumulated and saved in an additional volume. The manufacturer of the Aquilion CT system (Toshiba Medical Systems) disclosed two measured X-ray spectra (100 kV and 120 kV) and information about the design of the CT system. This information was implemented in the Monte Carlo simulation. A MatLab script (The MathWorks, Natick, MA, USA) was used to extract organ doses from the calculated dose distributions within the MIRD mathematical phantoms.

In CT it is common practice to adjust acquisition parameters to the size of the patient, and to the clinical application [33]. The acquisition protocol we used was based on local and optimised practices, and was checked against general recommendations, particularly with regard to the optimisation of the paediatric acquisitions. We thus derived the following clinical CT acquisition parameters. For small children, a tube voltage of 100 kV was used (weight <30 kg), and for larger children and adults a tube voltage of 120 kV (≥ 30 kg), both in combination with a pitch factor of 0.83. Radiation output of the CT system is expressed as the volume computed tomography dose index (CTDIvol), but also the tube charge (mAs) is provided for scans that are performed with an Aquilion 64 CT system (Toshiba Medical Systems, Japan) [34]. The clinically applied tube current, rotation time and pitch factor are associated with CTDIvols of 2.1 mGy (newborn, 3.6 kg, 27 mAs), 2.9 mGy (1 year old, 9.7 kg, 38 mAs), 4.1 mGy (5 years old, 19.8 kg, 53 mAs), 4.6 mGy (10 years old, 33.2 kg. 37 mAs), 7.1 mGy (15 years old, 56.8 kg, 58 mAs), and 9.5 mGy (adult, 74 kg, 77 mAs).

Dose calculations that were performed for the MIRD phantoms with the Monte Carlo software yielded organ doses, total body dose and effective dose according to ICRP 103. Dose assessment was performed for 12 CT acquisitions, i.e. both for whole-body CT (including neck, chest and abdomen), and for CT of the neck and chest only, in all six age categories. These doses were incorporated into the risk model, as described under “Risk assessment”. From these results, appropriate organ doses and the effective dose were derived for the year of diagnosis and treatment and for the following years of surveillance. If necessary, linear interpolation of dose values was performed to yield dose estimations at ages not included in the table.

### Radiation exposure from ^18^F-FDG PET

For assessment of organ dose and effective dose from ^18^F-FDG PET, published tables were used that provide information about organ dose and effective dose per MBq of administered ^18^F-FDG activity. The ICRP provides information for 1-, 5-, 10- and 15-year-old children, and for adults [35]. Ruotsalainen et al. [36] published tables for estimation of radiation dose to the newborn in ^18^F-FDG PET studies. For PET a dose of 3 MBq of ^18^F-FDG per kg body weight was assumed to be administered, based on the current state-of art imaging with integrated PET/CT systems (f.i. Biograph 40 TruePoint PET-CT, Siemens Medical Systems, Knoxville, TN, USA) . The five different age categories in the children being analysed in this study correspond to ^18^F-FDG doses of 10 MBq (newborn), 30 MBq (1 year old), 60 MBq (5 years old), 100 MBq (10 years old) and 170 MBq (15 years old). In adults, administration of 220 MBq ^18^F-FDG was assumed.

### Risk assessment

We performed risk assessment for five age categories of male and female paediatric patients diagnosed with HD (newborns, and children at the age of 1, 5, 10 and 15 years) and in three age categories for adult male and female patients diagnosed with DLBCL at the ages of 55, 65 and 75 years.

The BEIR VII excess relative risk (ERR) model was used for calculating radiation risk [37]. Chapter 12 of this BEIR report provides the equations and parameters for estimating organ-specific solid cancer mortality and leukaemia mortality. The ERR is expressed as a function of gender, absorbed organ dose, age at exposure and the attained age, and includes three organ-specific fit parameters. The organs are stomach, colon, liver, lung, breast, prostate, uterus, ovary, bladder, other organs and thyroid.

In the BEIR VII ERR model, the organ-specific excess risk for solid cancer mortality is expressed, relative to the gender- and age-dependent risk of the background cancer mortality for specific organs. This background is the naturally occurring mortality, not the mortality that is induced by radiation exposure during medical imaging. Data on the risk of naturally occurring cancer mortality depending on organ, gender and attained age were derived from ICRP Publication 103 (Euro-American cancer mortality rates by age and site) [38]. Subsequently, according to the BEIR VII model, an overall ERR function depending on organ dose, gender, age at exposure and attained age was calculated for male and female patients. Organ dose was incorporated into the risk model as a function of attained age; this was required because patients have different weights (and ages) and thus receive different associated radiation exposures during diagnosis, treatment and surveillance of malignant lymphoma. In addition to radiation risk, mortality rates that are typical for the young patient group with HD and the adult patient group with DLBCL could be taken into account, i.e. the overall 10-year survival for HD (94%) and 5-year survival for DLBCL (58%) [27–30].

Risk calculations were performed using life tables, and they were done with and without taking into account the disease-related mortality. Life tables (also called mortality tables) are used in demography for measuring and modelling population processes. A life table shows, for each age, what the probability is that a person of that age will die before his or her next birthday. They allow for the calculation of the fraction of radiation-induced deaths, the reduction of life expectancy and the survival rate. An essential component of the life table is age- and gender-dependent mortality; in this study three different sources of mortality were integrated in the life tables: the background, the radiation induced, and the disease-related mortality. The background mortality that is typical for the asymptomatic population (gender- and age-specific probability of dying) was derived for the European population at large based on the Eurostat database [39]. The radiation-induced, age-, gender- and dose-dependent overall mortality that was calculated with the BEIR VII model as described in the previous section, was also incorporated into the life tables. Finally, disease-related mortality, as mentioned in the previous section, can also be integrated in the life tables. Calculations were carried out with the life tables with and without taking into account the mortality risks that are typical for patients with malignant lymphoma (HD- and DLBCL-related mortality rates).

## Results

### Radiation exposure from CT and ^18^F-FDG PET

The radiation exposure from whole-body CT, from CT of the neck and thorax, and from whole-body ^18^F-FDG PET for children (HD) according to the five age categories and for adults (DLBCL) are presented in Table 2. Effective dose increased gradually with age from 5.5 mSv to 13.3 mSv (whole-body CT), and from 3.4 mSv to 7.9 mSv (neck-chest CT). For ^18^F-FDG PET examinations the variations in effective dose were smaller, ranging from 2.8 mSv to 4.3 mSv. In CT the highest organ doses were observed for lung and thyroid, and variations in organ dose were modest for organs lying entirely within the anatomical area examined. In PET the highest organ dose by far was observed for the bladder.

**Table 2:** Radiation exposure doses in mSv (total body dose [only CT], effective dose and organ doses) for children with HD according to the five age and weight categories and for adults with DLBCL, for a whole-body CT (*WB-CT*), a CT of neck and chest (*CT neck-chest*), and a ^18^F-FDG PET (*PET*) respectively

	0 years old / 3.6 kg			1 year old / 9.7 kg			5 years old / 19.8.2 kg			10 years old / 33.2 kg			15 years old / 56.8 kg			Adult / 74 kg		
	WB-CT	CT neck-chest	PET	WB-CT	CT neck-chest	PET	WB-CT	CT neck-chest	PET	WB-CT	CT neck-chest	PET	WB-CT	CT neck-chest	PET	WB-CT	CT neck-chest^a^	PET
Total body	4.8	2.6	n.a.	5.7	3.0	n.a.	6.9	3.3	n.a.	6.9	3.5	n.a.	9.0	4.4	n.a.	10.7	5.3	n.a.
Effective dose	5.5	3.4	3.8	6.6	4.0	2.8	8.3	4.5	3.0	8.6	5.1	3.6	11.0	6.2	4.3	13.3	7.9	4.2
Bone marrow	2.2	0.9	3.1	2.7	1.2	1.8	4.1	1.7	1.9	6.2	2.9	2.2	10.4	5.0	2.4	13.4	7.0	2.4
Stomach	6.2	4.6	2.3	7.5	5.0	2.0	9.3	4.0	2.1	9.4	5.5	2.2	11.8	5.0	2.4	13.9	7.3	2.4
Colon	6.0	0.5	2.2	7.1	0.5	2.2	8.6	0.3	2.4	8.7	0.4	2.7	10.8	0.2	2.9	12.7	0.3	2.9
Liver	6.2	4.6	4.4	7.4	5.2	2.0	9.1	4.6	2.2	9.1	6.1	2.2	11.2	6.3	2.4	13.0	8.5	2.4
Lung	6.7	6.5	2.2	8.1	7.9	1.9	10.1	9.7	2.0	10.1	9.9	2.1	12.4	12.1	2.4	14.8	14.5	2.2
Breast	5.6	5.5	2.3	6.6	6.3	1.6	8.4	8.0	1.7	8.1	8.1	1.8	9.1	9.0	1.9	11.1	11.0	1.9
Prostate	5.3	0.1	2.7	5.9	0.0	2.0	7.1	0.0	2.1	7.0	0.0	2.2	8.6	0.0	2.4	10.6	0.0	2.4
Uterus	6.1	0.3	4.0	7.3	0.2	2.9	9.0	0.1	3.3	9.1	0.1	3.9	11.6	0.1	4.4	14.4	0.1	4.6
Ovary	6.0	0.3	3.3	7.0	0.3	2.4	8.5	0.1	2.6	8.6	0.1	3.0	10.6	0.1	3.4	12.6	0.1	3.3
Bladder	6.0	0.1	11.1	7.2	0.1	17.2	9.0	0.0	19.0	9.3	0.0	27.9	12.2	0.0	35.8	15.3	0.0	35.4
Other solid organs	4.8	2.6	3.8	5.7	3.0	2.8	6.9	3.3	3.0	6.9	3.5	3.6	9.0	4.4	4.3	10.7	5.3	4.2
Thyroid	6.4	6.4	2.2	8.7	8.6	2.0	11.4	11.3	2.1	12.8	12.7	2.1	19.4	19.5	2.2	24.8	24.8	2.2

*n.a.* not available

^a^This acquisition is not part of the clinical protocols

Neonates and children with HD usually undergo seven whole-body CTs, five of the neck and chest, and two ^18^F-FDG PETs over a period of 5 years. Adults with DLBCL usually undergo seven whole-body CTs and one ^18^F-FDG PET during a period of 2.5 years. Because of the intensive use of X-ray imaging, the effective dose accumulates rapidly in patients with malignant lymphoma. In children with HD, cumulative effective dose reaches 66 mSv (newborn at diagnosis), 69 mSv (1 year old at diagnosis), 80 mSv (5 years old at diagnosis), 90 mSv (10 years old at diagnosis) and 113 mSv (15 years old at diagnosis). Differences between these paediatric age categories are due to differences in patient size and the patient size-specific acquisition protocols for both CT and ^18^F-FDG PET. In adults with DLBCL the cumulative effective dose reaches 97 mSv 2.5 years after diagnosis.

### Risk assessment

Demographic and risk-related parameters were derived from the life tables for neonates and children (with HD) corresponding to the five different age and weight categories. The paediatric categories are identified by the age at diagnosis (Table 3) and for adult patients (with DLBCL) the categories were 55, 65 and 75 years of age at diagnosis of the disease (Table 4).

**Table 3:** Risk assessment for five categories of children with HD based on a demographic methodology and life tables with and without correction for disease-related mortality. Quantification of cumulative patient radiation dose and associated mortality risk for patients with different ages at time of diagnosis

	0 years old				1 year old				5 years old				10 years old				15 years old			
	Male	Female	Male	Female	Male	Female	Male	Female	Male	Female	Male	Female	Male	Female	Male	Female	Male	Female	Male	Female
Corrected for disease-related mortality	no	no	yes	yes	no	no	yes	yes	no	no	yes	yes	no	no	yes	yes	no	no	yes	yes
Fraction radiation-induced deaths	0.005	0.010	0.003	0.007	0.006	0.011	0.004	0.007	0.006	0.011	0.004	0.007	0.006	0.011	0.004	0.007	0.007	0.012	0.005	0.008
Life expectancy at year of diagnosis (years)	76	82	60	64	76	81	60	63	72	77	58	61	67	72	54	58	62	67	51	55
Radiation-induced reduction of life expectancy (days)	29	64	19	41	30	68	20	43	32	71	21	46	32	68	22	46	35	74	25	51
Disease-related reduction of life expectancy (days)	n.a.	n.a.	5841	6560	n.a.	n.a.	5731	6440	n.a.	n.a.	5205	5881	n.a.	n.a.	4577	5213	n.a.	n.a.	3985	4578
Total reduction of life expectancy (days)	29	64	5859	6601	30	68	5750	6483	32	71	5226	5928	32	68	4598	5259	35	74	4009	4629
10-year survival after diagnosis (percentage)	99	99	93	94	100	100	94	94	100	100	94	94	100	100	94	94	99	100	93	94

*n.a.* not applicable

**Table 4:** Risk assessment for three categories of adults with non-Hodgkin’s lymphoma (NHL), subtype DLBCL, based on a demographic methodology and life tables with and without correction for disease-related mortality. Quantification of cumulative patient radiation dose and associated mortality risk for patients with different ages at time of diagnosis

	55 years old				65 years old				75 years old			
	Male	Female	Male	Female	Male	Female	Male	Female	Male	Female	Male	Female
Corrected for disease-related mortality	no	no	yes	yes	no	no	yes	yes	no	no	yes	yes
Fraction of radiation-induced deaths	0.004	0.005	0.001	0.001	0.003	0.004	0.001	0.001	0.002	0.002	0.001	0.001
Life expectancy at year of diagnosis (year)	25	29	9	9	17	20	7	8	10	12	6	6
Radiation-induced reduction of life expectancy (days)	14	23	2	2	9	13	2	2	4	5	1	1
Disease-related reduction of life expectancy (days)	n.a.	n.a.	5931	7228	n.a.	n.a.	3486	4354	n.a.	n.a.	1715	2057
Total reduction of life expectancy (days)	14	23	5933	7230	9	13	3488	4356	4	5	1716	2058
5-year survival after diagnosis (percentage)	96	98	58	59	90	95	55	58	76	86	46	52

*n.a.* not applicable

Calculations were performed with and without taking into account the disease-related mortality rates typical for the patient cohorts (Table 3). Risk assessment taking into account the effects of disease-related mortality resulted in a substantially lower fraction of radiation-induced deaths, and a lower associated reduction of life expectancy.

The calculations, which included disease-related mortality [or without disease-related mortality *in brackets*], resulted in an average 10-year survival of 94% (range 93–94%) [100% (range 99-100%)] for the young patient group with HD and an average 5-year survival of 55% (range 46–59%) [90% (range 76-98%)] for the adult patient group with DLBCL. Within the paediatric patient group, the average fraction of radiation-induced deaths was 0.4% [0.6%] for boys and 0.7% [1.1%] for girls. Within the adult group these values were even smaller; 0.07% [0.28%] for men and 0.09% [0.37%] for women. The average radiation-induced reduction of life expectancy in paediatric patients with HD is 21 days for boys and 45 days for girls. In adults with DLBCL, the average radiation-induced reduction of life expectancy is 1.5 days in men and 2.0 days in women.

## Discussion

This study shows that the effect of disease-related mortality on radiation risk assessment in patients with malignant lymphoma is substantial. By taking into account disease-related mortality, the proportion of radiation-induced deaths decreased between 30% and 40% in paediatric patients with HD, and between 50% and 80% in adults with DLBCL. Radiation-induced reduction of life expectancy decreased by similar percentages. Our methodology, which integrates a model for organ dose assessment and risk assessment in one demographic model, showed that the potential radiation-induced reduction of life expectancy is only a small fraction compared with the disease-related reduction of life expectancy, namely a fraction between 0.003 and 0.011 for HD (children); and between 0.0003 and 0.0006 for DLBCL (adults). This reflects the fact that the radiation-related risk is a late risk with its expression up to decades after the actual exposure and has therefore less effect on the reduction of life expectancy. Our methodology for radiation risk assessment is an improvement compared with the usual standard of practice, where disease-related mortality is not considered. Although the observed cumulative effective doses are relatively high for imaging patients with malignant lymphoma, the associated estimated radiation risks are still very modest.

Within the paediatric patient group, it is estimated that, on average, 0.4% of male and 0.7% of female patients eventually die because of the radiation exposure associated with medical imaging. For adult patients diagnosed with DLBCL, it is estimated that 0.07% of male and 0.09% of female patients die because of radiation exposure. The higher values for women result mainly from the relatively high sensitivity of the female breast to radiation exposure [40].

Calculation in which the disease-related mortality was taken into account resulted in an average 10-year survival of 94% (range, 93–94%) for the young patient group with HD, which matches excellently with the published overall 10-year survival of 94% [27, 28]. Similarly, for the adult patient group with DLBCL, the calculated average 5-year survival was 55% (range, 46–59 %), which matched well with the published value of 58% [29, 30]. The good agreement between our calculated reduction in survival after diagnosis and published values is a clear indication that risk assessment should be performed taking disease-related mortality into account.

This study has some limitations. Malignant lymphoma comprises a heterogeneous group of entities, with more than 40 subtypes [41]. In addition, with regard to radiation exposure, age is an important factor. Therefore we chose to analyse the risk of radiation exposure in only two types of lymphoma, HD in paediatric patients and DLBCL in adult patients (the most common malignant lymphoma subtypes in these age groups). Another limitation is that the assumed 10- and 5-year survival rates (for HD and DLBCL, respectively) may be an underestimation of the current situation, because treatment strategies have continued to evolve [27–30]. In this study, we focused on the mortality risk caused by the radiation exposure of the patient. The morbidity risk induced by the radiation exposure was not considered. It can be assumed that not all patients with a radiation-induced malignancy die from this malignancy, and more patients suffer from radiation-induced diseases than the number of patients expressed in the mortality risk. Our calculations are based on a methodology that is according to broadly accepted dosimetric techniques and risk models. However, radiation dose assessment and risk assessment at the low dose levels that are common in diagnostic radiology are always associated with considerable uncertainties, implying that the absolute risk figures that we calculated should be interpreted with care.

Furthermore, with the implementation of integrated PET-CT systems, low-dose CT is performed in addition to PET, as it is used for attenuation correction in PET imaging. The use of CT for attenuation correction reduces the examination time, which implies a lower dose of FDG (5 MBq/kg vs 3 MBq/kg). The low-dose CT was not included in this study. Two or one additional low-dose CT(s) would have been performed in children with HD and in adults with DLBCL, respectively, with each low-dose CT causing extra radiation exposure of only approximately 3 mSv for adults [42]. Finally, it should be recognised that there are considerable uncertainties in the radiation risk model of BEIR, especially regarding the risk at low-dose levels such as those encountered in CT [17].

In order to establish the most efficient imaging strategy and limit the radiation exposure in patients with malignant lymphoma, there are different options. It may be sufficient to use only the low-dose whole-body CT (combined with FDG-PET) during and after therapy instead of a diagnostic (i.e. full-dose) whole body CT. This implies a reduction in radiation exposure of 6 times 8 mSv for adults. In patients with a FDG-avid type of malignant lymphoma and a baseline ^18^F-FDG PET-CT combined with a diagnostic CT, the most accurate and appropriate method of imaging during follow-up may therefore prove to be the low dose PET-CT, without the diagnostic CT. Another recent development is the introduction of whole-body magnetic resonance imaging (MRI) for the evaluation of malignant lymphoma. Initial results on this application of whole-body MRI are promising, but more research is still needed before it can be recommended as an alternative to CT and/or ^18^F-FDG PET [43–46].

In conclusion, the disease-related reduction in life expectancy of patients diagnosed with malignant lymphoma must be taken into account to achieve more realistic estimates of radiation risk. It results in higher overall mortality and substantial lower incidence of radiation-induced deaths. Although the cumulative effective dose from medical imaging is high, the actual calculated radiation risks are very modest. The radiation exposure that results from imaging with CT and ^18^F-FDG PET is considered as justified in patients with malignant lymphoma, but should still be performed with care, especially in children. Ongoing studies have to establish the most efficient imaging strategies for (the different subtypes of) malignant lymphoma.
